# Average and Maximum Papilla Heights around Dental Implants in the Anterior Maxillary Region: A Retrospective Clinical Study

**DOI:** 10.1155/2022/4235946

**Published:** 2022-02-11

**Authors:** Gholam Ali Gholami, Soheil Hariri, Reza Amid, Leyla Roghanizadeh, Mahdi Kadkhodazadeh, Amirreza Mehdizadeh, Navid Youssefi

**Affiliations:** ^1^Periodontics Department, Dental School, Shahid Beheshti University of Medical Sciences, Tehran, Iran; ^2^Department of Prosthodontics, Faculty of Dentistry, Shahed University, Tehran, Iran; ^3^Dental Research Center, Research Institute of Dental Sciences, Dental School, Shahid Beheshti University of Medical Sciences, Tehran, Iran; ^4^Oral and Maxillofacial Radiology Department, Dental School, Islamic Azad University, Tehran Branch, Tehran, Iran; ^5^Prosthodontics Department, Dental School, Qazvin University of Medical Sciences, Qazvin, Iran

## Abstract

**Objective:**

The aim of this study was to determine the average and maximum height of the papilla around maxillary anterior implants in respect of neighboring structures and location of implants.

**Materials and Methods:**

92 dental implants from 63 patients were investigated in this study. Those implants were placed in the anterior maxillary region and had been loaded for a minimum of one year. After receiving written consent, clinical data including the height of interproximal papillae adjacent to the tooth/implant/pontic were obtained through clinical observation. The independent *t*-test or ANOVA, the regression modeling, and generalized estimating equation (GEE) models were used for statistical analysis (*p* < 0.05).

**Results:**

Papilla height was calculated as 2.8 mm (1–5.5 mm) for implant-tooth sites, 2.6 mm (1–4 mm) in implants beside pontics, and 2.5 mm (1–3.5 mm) for implants adjacent to implants. Despite the lack of a significant difference in the mean papilla height in the studied groups, the maximum values of papilla heights were significantly different.

**Conclusions:**

In this study, no significant differences were found in papilla height mean values in relation to neighboring structures or location of implants in the anterior maxilla. However, the maximum values of papilla heights were observed around implants next to natural teeth.

## 1. Introduction

Up to one-quarter of adults in Western countries have lost at least one anterior tooth despite a decline in the prevalence of tooth loss [1]. Implant-supported crowns are the best known treatment for rehabilitating the missing teeth [2]. However, establishment of favorable esthetic and achieving harmony among crowns supported by implants and adjacent natural teeth are a formidable trial while reconstructing the edentulous space in the anterior maxilla [3]. Setting the implant soft-tissue architecture in accordance with the adjacent teeth/implants and periodontium is still a challenge in implant dentistry [4]. The height of the papilla between a single-tooth implant and the adjacent teeth and the peri-implant soft-tissue filling between two adjacent implants are among the crucial determining factors in this challenge [5].

Loss of papillary height and consequent open embrasures endanger desirable esthetic needed in the anterior region and may create obstacles to maintain supporting tissues' health. Therefore, a better understanding of associated factors is necessary in order to prevent this problem [6]. The relative tooth position and shape [7], the morphology of the interproximal space [8], the distance between the contact point and the alveolar crest [9, 10], the amount of keratinized tissue [10], gingival thickness and biotype [10], the implant position [10], the osseous crest location [7] and thickness, especially the thickness of the labial plate [11], and different surgery procedures [10, 12] are important key points which clinicians need to consider in order not to have implant soft-tissue complications [10]. Moreover, implant materials can be influential as papillae filling the entire interproximal space and their esthetic scores were more and better beside zirconia implants than titanium ones [13, 14]. Furthermore, good scores in the papilla index and other esthetic scores can be obtained regardless of either immediate or delayed loading [2].

Although the presence and stability of the interdental papilla adjacent to the implant are associated with many factors, the role of each factor and their interaction with each other are unclear yet [15]. Thus, despite the above studies, complete information about the factors and conditions that are associated with hard‐ and soft‐tissue deficiencies at implant sites is still needed, and further studies to better identify the associated factors should be taken into account [16].

The aim of this retrospective clinical study was to determine the mean and maximum values of peri-implant papilla height in the anterior maxillary region and to investigate whether there could be an association between height of peri-implant papillae with different adjacent structures to which the implant was in contact with (the implant-implant, implant-tooth, or implant-pontic) and/or implantation location (the central or lateral incisors, canine, or first premolar). The null hypothesis could be no impact of different adjacent structures or implantation location on height of implant interproximal papillae in the anterior maxilla. However, in fact, our study had an experimental hypothesis that evaluated the effect of some local factors on the papilla height of implants in the anterior maxillary region.

## 2. Materials and Methods

This was a descriptive cross-sectional study which was approved by the ethics committee of Shahid Beheshti University of Medical Sciences (IR.SBMU.RIDS.REC.1394.144). The study had been conducted in full accordance with the World Medical Association Declaration of Helsinki. The samples were collected through convenience sampling from the patients who were treated in two private clinics in Tehran from their records. All the patients signed an informed consent form. The inclusion criteria were the age of 25 to 80 years, having at least one implant in the anterior maxillary region, passing at least one year from the loading time, full mouth plaque index lower than 20%, and following a regular maintenance program. The exclusion criteria were diabetic patients with poor glycemic control, having a history of smoking, plastic surgery in the anterior maxillary region, radiotherapy of the head and neck, using drugs that could possibly induce gingival hyperplasia or interfere with bone healing (such as parenteral bisphosphonates), and evidence of hyperplastic inflammation, peri-implantitis, or bone loss in parallel periapical views. For every patient, the study procedure was explained, and if the patient was contented to participate in the study, written consent was obtained. Then, for each participant, a questionnaire including background information (age, sex, smoking status, systemic diseases, and medication), implant system, surgical method, location of implantation (the central or lateral incisors, canine, or first premolar), whether the inserted implant was adjacent to the tooth/implant/pontic, the materials and methods used for replacing missing teeth, and suprastructure data (abutment and prosthesis type, bone level, or tissue level) were acquired. Patients with poorly controlled type 1 diabetes mellitus (HbA1c > 7%) and smoker patients were excluded.

To ensure that the patient had observed and considered the minimum plaque control needed to survive the implant, oral hygiene status was evaluated based on the presence/absence of visible plaque at the soft-tissue margin of the four sites by a trained general dentist. Then, a calibrated periodontist determined the clinical parameters with a periodontal probe (UNC-15 Williams probe; Hu‐Friedy, Chicago, IL, USA) according to the study of Fischer et al. [17]. The papilla height of single/multiple adjacent implants was measured as the distance between coronal edges of the interdental papilla and the line connecting the midfacial soft-tissue margin of two adjacent implant-tooth/implant/pontic [17]. The width of keratinized soft tissue was measured with the periodontal probe as the distance between the soft-tissue margin and the mucogingival line measured midfacially to the nearest 0.5 mm [17].

The collected data were entered into Excel 2013 software (Microsoft Corporation, Redmond, WA, USA). Data analysis was performed using independent *t*-test or ANOVA. If one patient had more than one implant, we considered those implants independent. Moreover, regression modeling and generalized estimation equation (GEE) were used to detect the significance of differences and correlations. GEE analysis was performed by the same method as Chang and Wennström used in their study [18].

## 3. Results

We first reviewed the files of 78 patients. These patients had 119 loaded implants and 238 contact areas in the anterior maxillary region. Each studied patient had at least one implant that replaced the maxillary central incisor, lateral incisor, canine, or first premolar tooth (location 1, 2, 3, or 4, respectively). None of the patients had a history of head and neck radiotherapy or medication that could possibly induce gingival hyperplasia or interfere with bone healing (such as parenteral bisphosphonates). None of the patients had any evidence of hyperplastic inflammation, peri-implantitis, or bone loss in parallel periapical views. Nine patients were excluded from the study due to type 1 diabetes or smoking. Five patients did not cooperate for follow-up appointments. All patients had good oral health, and none of them had visible plaque. Only two implants of one patient (about 2% of samples) were of tissue level, and the rest were bone-level implants; thus, those two tissue-level implants were excluded from our study.

Finally, 184 contact areas (92 implantation sites) in 63 patients were included in the study. They included 34 female and 29 male patients. The mean age of patients at the time of implant placement was 52.0 ± 12.2 years. All the implants had 3 to 4.5 mm diameters and 10 to 17 mm lengths.

**Figure 1:**
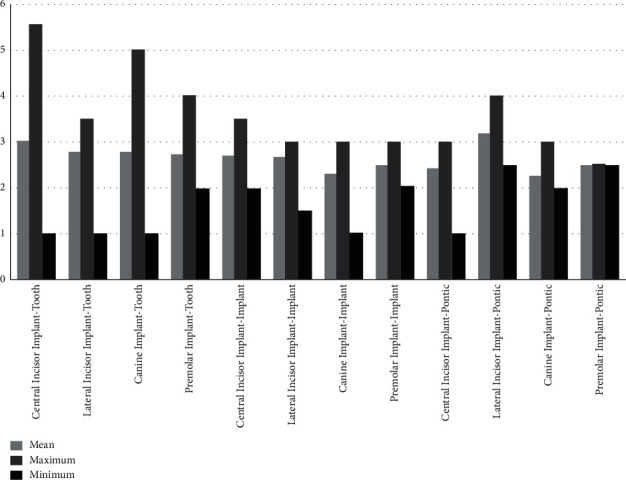
Bar diagram of the mean, minimum, and maximum of the implant papillary heights in different tooth locations with different contacts. The vertical axis represents heights of papillae in millimeter.

Mean, minimum, and maximum of the papilla height in different contact area groups and different locations are obtained and shown in Figure 1, and the associated data are presented in Table 1. In the implant-tooth group, the papilla height was 2.87 ± 0.75 mm (1.0–5.5 mm). Height in the implant-implant group was calculated as 2.50 ± 0.61 mm (1.0–3.5 mm). In the implant-pontic group, the height was computed as 2.65 ± 0.66 mm (1.0–4 mm). There was no significant difference in the mean value of papilla height among these groups. However, the maximum height of the papilla was higher in the implant-tooth group in comparison with implant-implant and implant-pontic sites.

The data of papilla height of the three contact groups, concerning different locations where the implants had been inserted, are presented in Table 2. Contact at the central incisor was considered as the reference. As the results of a regression analysis, in the implant-tooth group, there was no significant difference of the measured papilla height between the central incisor location and other locations (the lateral incisor, canine, or first premolar). Similarly, in the implant-implant group and implant-pontic group, papilla height in different locations (the lateral incisor, canine, or first premolar) did not differ significantly from the central incisor location.

Furthermore, according to the analysis of variance (ANOVA), the difference in papillary height with regard to contact groups (implant-tooth, implant-implant, and implant-pontic) was not significant (mean sum of squares = 1.440, *F* = 2.748, *p*=0.067 (*p* > 0.05)).

Using linear regression to model a relationship between two sets of variables presented the significant relation of age and peri-implant papilla height (95% confidence interval = _0.024 to _0.002, *p*=0.019 (*p* < 0.05)). Calculation of the correlation coefficient showed a reverse relation between patients' age and papilla height around implants (*r* = −0.219), and data scatter rate from the age of 25 to 80 years had led to a stronger correlation. On the contrary, the linear regression showed no significant relation between keratinized tissue width and peri-implant papilla height (95% confidence interval = _0.145 to 0.117, *p*=0.834 (*p* > 0.05)), and also, the correlation between these variables was shown to be very weak (*r* = −0.014).

Multivariable regression modeling and generalized estimation equation (GEE) of papilla height and assessed variables were also carried out as shown in Table 3. No significant relationship between the papilla height and width of keratinized tissue, the patients' gender, and the implantation location could be detected. Again, the only traceable correlation was the inverse association between age of the patient and papilla height (df = 1, *B* = −0.012, standard error = 0.005, *p*=0.023 (*p* < 0.05)). No other correlations could be noticed.

## 4. Discussion

To be able to obtain a suitable interproximal papilla while reconstructing an extracted tooth with an implant has been indicated as a difficulty to achieve in several studies [8]. In the present study, we assessed the association between three different implant contact groups (implant-tooth, implant-implant, and implant-pontic) and peri-implant papilla height. Also, we analyzed the relationship of four different locations of implant placement in the maxillary anterior region (the central incisor, lateral incisor, canine, and first premolar) with the implant papilla height. No statistically significant differences were disclosed between the mean values of the papilla heights among three different implant contact groups, and also, papilla heights showed no significant difference among four different implant locations.

Contrary to our results, in the study of Agabiti et al., assessment of mean index scores of papillae based on the adjacent element showed highest mean scores in interdental embrasures located between two implants or implants and pontics, which had statistically significant differences [19]. On the contrary, Agra Souza et al., in their study, observed that, in all implant-implant contact areas, complete papilla was not formed, indicating that papillary formation becomes more difficult when there is no natural dental structure [8]. Moreover, in the study of Cosyn et al., comparing different areas of contact, papilla formation between two adjacent implants had poorer results, and they concluded that clinicians should expect the formation of a short papilla and less satisfactory implant-supported restorations in these situations [20].

In our study, despite no significant difference in three different implant contact groups, statistically significant differences were observed between the maximum values of papilla height in contact groups. The highest values of papilla height were observed in the implant-tooth group and then in the implant-pontic group, and the lowest values of papilla height belonged to cases of the implant-implant group.

In this study, we could not find any correlation between the papilla height and the width of keratinized gingiva. A very recent systematic review by Vlachodimou et al. concluded that the width of keratinized gingiva seems to have a positive correlation with the periodontal phenotype. In addition, their results emphasized that the width of keratinized gingiva and periodontal phenotype can influence the outcome of periodontal and restorative treatments [21]. Furthermore, a recent study conducted by Garabetyan et al. considered periodontal biotype and the height of both keratinized tissue and the neighboring papilla as the crucial factors which preserve the peri-implant soft tissues' stability [15].

In the present study, no significant association was spotted between the location of implants anteroposteriorly (from the central incisor to first premolar) and papilla height. Our results were in agreement with the results of Schropp and Isidor study [22], in which the implant location (anterior vs. posterior) did not have a significant influence on the papilla height. Nevertheless, Kolte et al. [23] concluded that the apicocoronal proximal contact area (from the apical most point of contact area to the coronal most incisal point) gradually decreases from maxillary central incisors to first premolars in a distal direction [23].

A significant inverse relation between patients' age and the height of papilla around the implant was found in the results of the present study. Our results were in agreement with the recent study of Kolte et al. [23], that greater bulks of the papilla were observed in the group of younger patients. Also, in agreement with these results, in the randomized clinical trial of Schropp and Isidor [22], younger patients had significantly better papilla scores than older patients.

The retrospective nature of this study along with different sample sizes in each group would be considered as the main limitations. It would be better to perform future studies not only with larger population but also with equal or similar number per group. Moreover, applying other examinations such as radiographic evaluations to measure the position of the contact point in relation to the level of the bone crest is needed to prove the effect of different influential factors on the height of the implant papilla. Also, it is recommended to perform studies with longer-term follow-ups to evaluate alterations in peri-implant papilla height over a longer period of time.

## 5. Conclusion

According to the results of this study, among different implant contact groups (implant-tooth, implant-implant, and implant-pontic) or different locations (the central incisor, lateral incisor, canine, or premolar), no significant difference in the average values of papilla height could be detected. However, the maximum height of the papilla could be observed in the implant-tooth group. In addition, there was an inverse correlation between patient's age and papilla height.

## Figures and Tables

**Table 1:** Number of contact areas in three contact area groups and four locations and mean, standard deviation (SD), minimum (MIN), and maximum (MAX) of the papilla height in each group.

Group	Location	Numbers of contact areas	Mean ± SD (mm)	MIN (mm)	MAX (mm)
1. Implant-tooth	Central incisor	46	3.033 ± 0.819	1	5.5
	Lateral incisor	28	2.786 ± 0.659	1	3.5
	Canine	50	2.820 ± 0.740	1	5
	First premolar	16	2.719 ± 0.682	2	4
	Total	140	2.868 ± 0.748	1	5.5
2. Implant-implant	Central incisor	8	2.688 ± 0.530	2	3.5
	Lateral incisor	6	2.688 ± 0.544	1.5	3
	Canine	4	2.250 ± 1.000	1	3
	First premolar	3	2.500 ± 0.500	2	3
	Total	21	2.500 ± 0.612	1	3.5
3. Implant-pontic	Central incisor	8	2.438 ± 0.728	1	3
	Lateral incisor	8	3.188 ± 0.458	2.5	4
	Canine	6	2.250 ± 0.418	2	3
	First premolar	1	2.500 ± 0	2.5	2.5
	Total	23	2.652 ± 0.664	1	4

**Table 2:** Results of regression analysis: significance level of papilla height mean in (A) implant-tooth, (B) implant-implant, and (C) implant-pontic areas separately by location ((1) central incisor, (2) lateral incisor, (3) canine, and (4) first premolar).

Groups	Location	*B* ^*∗*^	Standard error	95% confidence interval (CI)		*p* value
				Lower	Higher	
Implant-tooth	1. Central incisor	0.000	0.000	0.000	0.000	_
	2. Lateral incisor	−0.256	0.201	−0.659	0.129	0.188 (*p* > 0.05)
	3. Canine	−0.213	0.170	−0.547	0.121	0.212 (*p* > 0.05)
	4. First premolar	−0.314	0.242	−0.788	0.161	0.195 (*p* > 0.05)
Implant-implant	1. Central incisor	0.000	0.000	0.000	0.000	_
	2. Lateral incisor	−0.436	0.324	−1.012	−0.334	0.136 (*p* > 0.05)
	3. Canine	−0.188	0.476	−1.122	−0.747	0.694 (*p* > 0.05)
	4. First premolar	−0.188	0.324	−0.823	−0.448	0.563 (*p* > 0.05)
Implant-pontic	1. Central incisor	0.000	0.000	0.000	0.000	_
	2. Lateral incisor	0.750	0.397	−0.028	1.528	0.059 (*p* > 0.05)
	3. Canine	−0.188	0.241	−0.661	0.286	0.438 (*p* > 0.05)
	4. First premolar	—	—	—	—	—

*B* ^*∗*^: difference between the papilla heights in the mentioned location compared to that of the central incisor location in each group.

**Table 3:** Regression modeling and generalized estimation equation (GEE) of papilla height and assessed variables.

Parameter	*B*	Standard error	95% Wald confidence interval		Hypothesis testing		*p* value
			Lower	Upper	Wald chi-square test	df (degree of freedom)	
Intercept	3.834	0.520	2.813	4.855	54.180	1	(*p* < 0.001)
Keratinized tissue width	−0.0.10	0.077	−0.161	0.142	0.016	1	0.899 (*p* > 0.05)
Gender 1 (male)	0	—	—	—	—	—	_
Gender 2 (female)	−0.061	0.118	−0.294	0.172	0.266	1	0.606 (*p* > 0.05)
Central incisor location	0	_	_	_	_	_	_
Lateral incisor location	−0.165	0.171	−0.501	0.171	0.926	1	0.336 (*p* > 0.05)
Canine location	−0.208	0.142	−0.488	0.072	2.111	1	0.146 (*p* > 0.05)
Premolar location	−0.245	0.199	−0.635	0.146	1.509	1	0.219 (*p* > 0.05)
Implant-tooth contact	0	—	—	—	—	—	_
Implant-implant contact	−0.298	0.174	−0.639	0.044	2.919	1	0.088 (*p* > 0.05)
Implant-pontic contact	−0.139	0.213	−0.557	0.279	0.426	1	0.514 (*p* > 0.05)
Age	−0.012	0.005	−0.021	−0.002	5.177	1	0.023 (*p* < 0.05)
Scale	0.519	—	—	—	—	—	—

## Data Availability

Raw data and derived data supporting the findings of this study are available from the corresponding author upon request.
